# Primary Pulmonary Malignant Fibrous Histiocytoma

**DOI:** 10.1155/2015/381276

**Published:** 2015-03-08

**Authors:** Devin P. Patel, Yogesh S. Gandhi, Keith E. Sommers, Devanand Mangar, Enrico M. Camporesi

**Affiliations:** Tampa General Hospital, Tampa, FL 33606, USA

## Abstract

Malignant fibrous histiocytoma (MFH) is one of the most common adult soft tissue sarcomas. MFH is very aggressive and is most often found in the extremities and the retroperitoneum, but it can manifest at other sites. Though the lungs are the most common sites of metastasis, they rarely present there as a primary tumor. Our report describes a rare case of a primary MFH tumor in the lung. Careful diagnostic procedure should be followed to ensure the tumor does not have extrapulmonary origins. Though MFH is highly invasive and deadly, surgical excision of the tumor has been shown to be successful.

## 1. Introduction

Malignant fibrous histiocytoma is an aggressive soft tissue sarcoma originating from mesenchymal cells containing both fibroblasts and histiocytes [1]. MFH is one of the most common soft tissue sarcomas of adulthood and typically presents between the ages of fifty and seventy. Although these cancers can occur anywhere in the body, they are most commonly found in the extremities and retroperitoneum [2]. These tumors have a high propensity for metastasis with the lungs being the most common site for distant metastasis. Although MFH is most commonly found in the lungs as a metastatic lesion, it can in rare instances present as a primary lung malignancy. Since the first reported case of primary pulmonary MFH in 1979, there have been approximately fifty additional reported cases in the English literature [3, 4]. Our case report of this extremely rare malignancy adds to the current English literature.

## 2. Case Report

**Figure 1:**
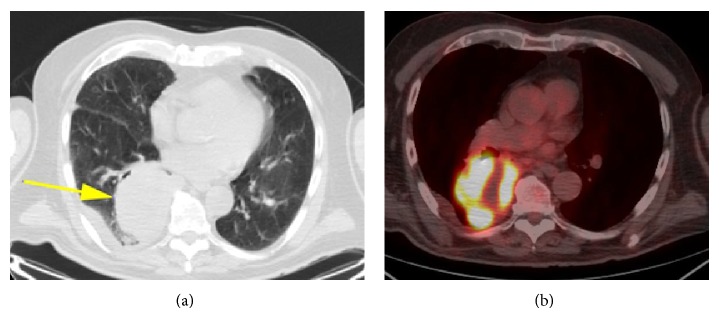
(a) Large right pleural mass indicated by the arrow. (b) Intense PET dye reaction indicates presence of metabolically active mass; uptake in the core of the lesion is reduced, reflecting tissue necrosis, verified upon surgical resection.

An eighty-six-year-old gentleman presented to his primary care physician with chief complaints of cough, dyspnea, and increasing weakness. His primary care physician ordered a chest X-ray which demonstrated a large mass in the right lung. Subsequently, a CT scan of the chest was ordered and demonstrated a large pleural-based cavitary lesion in right lower lobe measuring 8.2 × 7 cm (Figure 1(a)). A small pleural effusion was also noted. A PET/CT was performed which revealed a metabolically active solitary pulmonary mass with an elevated SUV of twenty-one (Figure 1(b)). There was no evidence of regional or distant metastases on imaging.

A CT guided lung biopsy was performed with pathology revealing an undifferentiated lung carcinosarcoma. Special stains were ordered and the tumor was negative for pancytokeratin, TTF 1, cytokeratins 7 and 20, epithelial membrane antigen, prostate specific antigen, carcinoembryonic antigen, HMB-45, S-100 protein, smooth muscle actin, and desmin. The tumor was positive for vimentin and focally positive for CD68 and P63. High proliferative activity was seen with Ki-67 being positive in 80% of cells. This tumor morphology was highly suggestive but not diagnostic of pleomorphic sarcoma. However, it was felt that highly undifferentiated carcinoma could also have similar characteristics. As a result, the specimen was sent to Biotheranostics for cancer type ID which uses a real-time RT-PCR platform, the “gold” standard for gene-expression. The Biotheranostics assay demonstrated a greater than 90% probability of the tumor representing a sarcoma.

**Figure 2:**
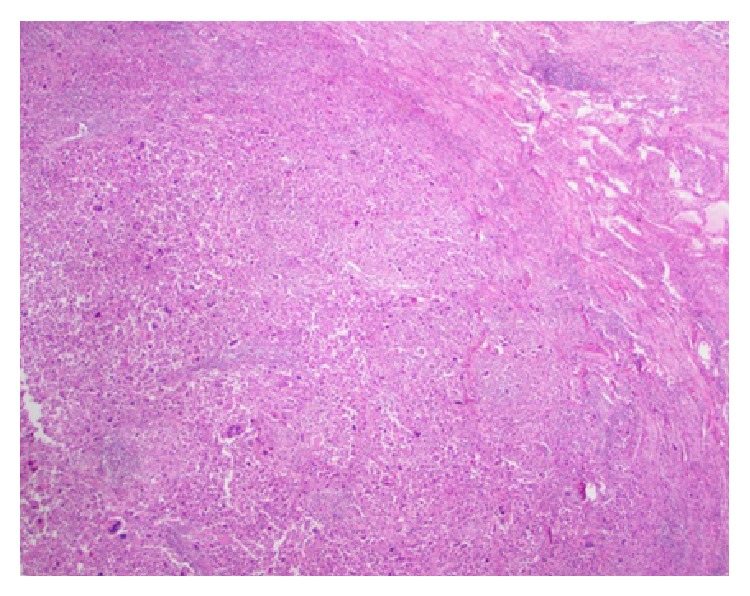
Hematoxylin and eosin stained section at 400x magnification demonstrating highly pleomorphic cells with prominent nucleoli in a storiform pattern. Multinucleated forms are also present.

**Figure 3:**
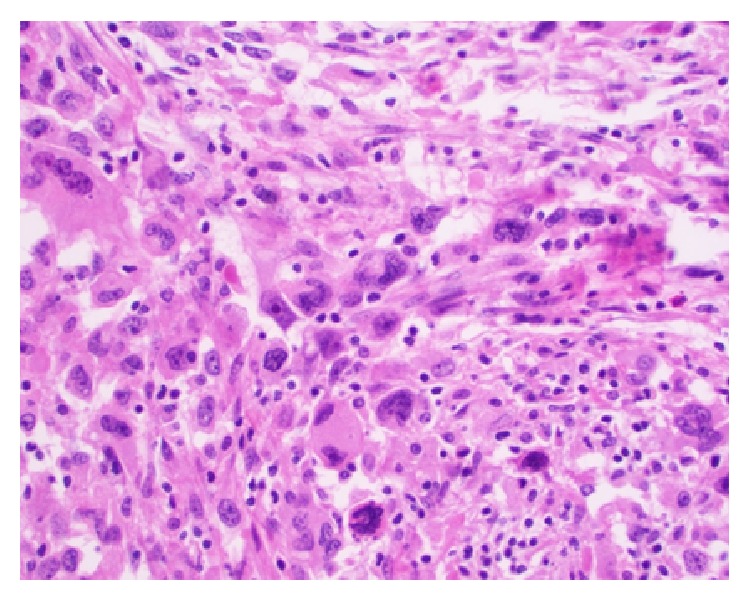
High power H & E stain photomicrograph demonstrating pleomorphic giant cells, spindle shaped fibroblasts, and histiocytes.

The patient was referred to thoracic surgery, as this was felt to provide the best chance for cure. The patient subsequently underwent flexible fiberoptic bronchoscopy with washings and right posterior thoracotomy. A right middle and lower lobe bilobectomy with mediastinal lymph node dissection was performed. Final pathology noted a tumor size of 9.6 × 8.9 × 7.6 cm and confirmed the diagnosis of a pulmonary pleomorphic high grade carcinosarcoma, malignant fibrous histiocytoma (Figures 2 and 3). All surgical margins and mediastinal lymph nodes were negative for tumor. The Enzinger-Weiss classification can be described as pleomorphic sarcoma [2]. Immunohistochemical stains revealed the tumor to be positive for vimentin. The patient recovered well from his surgery and is without evidence of disease six months later. The patient did not receive any adjuvant chemotherapy or radiation therapy, as both were considered ineffective in this type of tumor.

## 3. Discussion

As with our case presentation, primary pulmonary MFH appears to occur more frequently in elderly males; however, it can also present in women and in children [5, 6]. Presenting symptoms typically include cough, chest pain, and dyspnea [6, 7]. Other less frequent presenting symptoms include hemoptysis, fatigue, and weight loss. Rarely patients may be asymptomatic at presentation [7].

Radiographically, Reifsnyder et al. noted that seventy-five percent of patients with MFH of the lung presented with a solitary pulmonary nodule on imaging. Bilateral pulmonary nodules and pleural effusions were noted in ten percent of patients [8]. On computed tomography (CT) these tumors generally appear as soft tissue density lesions with or without central areas of attenuation [9]. CT of the chest also helps determine mediastinal involvement or subdiaphragmatic extension, as locoregional spread is not uncommon. We believe positron emission tomography (PET) imaging can be of great utility in ruling out a primary source elsewhere in the body, particularly the retroperitoneum.

Microscopically, MFH is composed of spindle shaped fibroblasts and histiocytes with atypical pleomorphic giant cells in varying proportions [10]. Five distinct histologic subtypes have been described: storiform-pleomorphic, myxoid, giant cell, inflammatory, and angiomatoid [2, 11]. The most common histologic subtype noted in primary pulmonary MFH is storiform-pleomorphic [6]. The marked cellular pleomorphism and atypia along with numerous mitotic figures found in primary pulmonary MFH distinguish it from benign fibrous histiocytic tumors of the lung which have also been reported [12–14].

Specific immunohistochemical stains for MFH do not exist. However, other sarcomas with similar microscopic findings can be excluded with immunohistochemical staining. As a result, stains for desmin, actin, vimentin, keratin, and neurogenic tumors are commonly obtained [6].

The prognosis for patients with primary pulmonary MFH in general is poor, yet survival in these patients can be variable. Our review of the literature generated 55 other reported cases (see Table 1) with survival in excess of 10 years [3–34]. Most patients were treated with surgical resection. These malignancies are aggressive and demonstrate a propensity for both local recurrence and distant metastasis. Nevertheless there are several reports of patients with long term survival [6, 15]. In fact, survival after complete surgical excision with clear margins for primary pulmonary MFH is reported to be better than for other pulmonary sarcomas [15]. As would be expected, a poorer prognosis is noted in those with advanced stage disease, incomplete resection, tumor invasion of the mediastinum or chest wall, recurrence, and presence of metastasis, thus underlining the importance of early diagnosis and surgical treatment [16].

Complete surgical resection with clear margins is the mainstay of treatment and yields the best chance for long term survival. Radiation therapy and chemotherapy in primary pulmonary MFH are generally considered ineffective. Systemic chemotherapy has typically been reserved for patients with metastatic disease. The role of radiation therapy is uncertain; however, some studies have advocated adjuvant radiotherapy ([6, 16] and Table 1).

Close follow-up is important as local recurrence is common and early metastasis particularly to the brain is not uncommon [12, 17, 18]. The propensity of these tumors for metastasis is postulated to be due to their high incidence of vascular invasion, which was noted in as many as fifty percent of specimens in one study [19].

In conclusion, primary pulmonary MFH is a rare malignancy with several reported cases. As metastatic MFH lesions are more commonly noted in the lungs, patients suspected with a primary pulmonary MFH should undergo a comprehensive diagnostic workup to rule out an extrapulmonary primary origin. Although these tumors are believed to be highly aggressive with a relatively high mortality rate and a relatively high incidence of local recurrence and distant metastasis, long term survival is possible and has been reported in several studies. The most widely accepted treatment is complete surgical excision with clear margins.

## Figures and Tables

**Table 1:** References, case listing, and treatment, comprising follow-up survival.

Reference	Age	Sex	Site	Size (cm)	LN	Treatment	F/U (mos)
Bedrossian et al. [3]	51	M	LLL/RML	2/4	NEG	Lobectomy	DOD (14)

Kern et al. [18]	53	M	RLL	8	NEG	Lobectomy	DOD (12)

Chowdhury et al. [21]	52	F	RLL	5	UNK	Chemotherapy	DOD (4)

Tsangaridou et al. [5]	65	F	RML	11.5	NEG	Pneumonectomy/chemotherapy	DOD (5)

Paulson et al. [22]	53	F	LLL	4	NEG	Lobectomy	DOD (36)

Mills et al. [23]	60	F	RLL	10	NEG	Lobectomy	AWD (18)

Sriumpai et al. [24]	41	M	RLL	9	UNK	Lobectomy	DOD (18)

Misra et al. [25]	45	M	RLL	16	POS	XRT	DOD (10)

Larsen et al. [26]	75	M	RUL	2.5	NEG	Wedge resection	NED (10)

	62	M	LLL	6	NEG	Lobectomy	NED (12)
	54	M	LUL	7	NEG	Chemotherapy	DOD (7)
Lee et al. [27]	69	M	RUL	8	NEG	Pneumonectomy/XRT	NED (8)
	62	F	LLL	5	NEG	Lobectomy/XRT	NED (120)
	67	M	LUL	4	NEG	Lobectomy	NED (60)

Lessel and Erbstösser [28]	35	F	RLL	25	UNK	None	DOD (12)

Silverman and Coalson [29]	56	M	LUL	8	NEG	Chemotherapy	AWD (3)

Tanino et al. [30]	75	M	LUL	5	POS	None	DOD (5)

McDonnell et al. [10]	73	F	LLL	6.5	NEG	Lobectomy	DOD (3)

Hsiu et al. [31]	71	F	RUL	5	NEG	Lobectomy	NED (10)

Juettner et al. [32]	58	M	RLL	5.5	POS	Lobectomy	DOD (12)
	68	M	LLL	20	NEG	None	DOD (12)

Ismailer et al. [33]	12	F	RUL	9	NEG	Lobectomy	AWD (12)

	54	F	RLL	1.7	NEG	Lobectomy	NED (108)
	33	M	RUL	3.8	NEG	Lobectomy	NED (84)
	59	M	RLL	5.9	NEG	Lobectomy	NED (65)
	73	F	LUL	8.5	POS	Pneumonectomy	NED (36)
	64	M	RUL	5	NEG	Lobectomy/XRT	NED (16)
	42	F	LLL	3	NEG	Lobectomy	NED (122)
	57	F	RUL	4	NEG	Pneumonectomy	DNED (1)
	80	M	LUL	3	NEG	Lobectomy	DNED (1)
	74	M	LUL	UNK	NEG	None	DOD (2)
	18	M	RLL	10	NEG	Lobectomy	DOD (1)
Yousem and Hochholzer [7]	46	F	RUL	6	POS	Lobectomy/XRT	DOD (8)
	52	F	RLL	UNK	NEG	Chemotherapy	DOD (9)
	52	F	LUL	4	POS	Lobectomy/chemotherapy/XRT	DOD (72)
	74	F	RUL	14	POS	Lobectomy	DOD (24)
	69	F	RUL	8	NEG	XRT	DOD (36)
	40	F	LLL	4	NEG	Lobectomy/XRT	DOD (24)
	74	M	RML	UNK	NEG	XRT	DOD (8)
	19	M	LUL	UNK	NEG	Lobectomy/chemotherapy/XRT	DOD (14)
	63	M	LLL	7	NEG	None	DOD (14)
	36	M	RLL	3	NEG	Excisional biopsy	DOD (12)
	32	M	LLL	11	POS	Pneumonectomy/chemotherapy/XRT	DOD (3)

Casey and Peddle [34]	21	M	RUL	3	NEG	Lobectomy	NED (96)
	46	M	LLL	10	NEG	Lobectomy	NED (8)

Palmer et al. [19]	62	F	RLL	UNK	NEG	Lobectomy	DOD (14)

White et al. [20]	55	M	RUL	UNK	UNK	None	DOD (4)

Mills et al. [23]	49	M	RML/RLL	5.5	NEG	Lobectomy	UNK

Marchan and Perez [4]	10	F	LLL	5	NEG	Lobectomy	UNK

Halyard et al. [6]	51	F	LLL	12	NEG	Lobectomy/XRT	NED (60)
	77	M	RML	2.2	NEG	Lobectomy	NED (36)
	38	M	LLL	11	NEG	Excisional biopsy	DOD (30)
	57	M	LUL	7.5	POS	Lobectomy	DOD (1)

Tsangaridou et al. [5]	UNK	M	LLL	UNK	NEG	Pneumonectomy	AWD (168)

Current Case	86	M	RML/RLL	9.6	NEG	Lobectomy	NED (6)

M = male; F = female; LLL = left lower lobe; RUL = right upper lobe; RML = right middle lobe; RLL = right lower lobe; UNK = unknown; NEG = negative; POS = positive; XRT = radiation therapy; DOD = dead of disease; NED = no evidence of disease; DNED = dead, no evidence of disease; AWD = alive with disease.
